# Gravity determines the direction of nerve roots sedimentation in the lumbar spinal canal

**DOI:** 10.1186/s12891-021-04032-y

**Published:** 2021-02-08

**Authors:** Jun Yang, Zhiyun Feng, Nian Chen, Zhenhua Hong, Yongyu Zheng, Jiang Yang, Tingjie Zhou, Xin Yao, Taifeng Xu, Linting Zhang

**Affiliations:** 1 Department of Orthopedic Surgery, Sanmen People’s Hospital, Taizhou, China; 2 Department of Orthopedic Surgery, The First Affiliated Hospital, Zhejiang University School of Medicine, Hangzhou, China; 3 Department of Orthopedic Surgery, Taizhou Hospital, Taizhou, China; 4 Operating room, Sanmen People’s Hospital, Taizhou, China

**Keywords:** Lumbar spinal stenosis, Gravity, Prone position magnetic resonance imaging, Nerve roots sedimentation sign

## Abstract

**Objectives:**

To investigate the role of gravity in the sedimentation of lumbar spine nerve roots using magnetic resonance (MR) imaging of various body positions.

**Methods:**

A total of 56 patients, who suffered from back pain and underwent conventional supine lumbar spine MR imaging, were selected from sanmen hospital database. All the patients were called back to our hospital to perform MR imaging in prone position or lateral position. Furthermore, the sedimentation sign (SedSign) was determined based on the suspension of the nerve roots in the dural sac on cross-sectional MR images, and 31 cases were rated as positive and another 25 cases were negative.

**Results:**

The mean age of negative SedSign group was significantly younger than that of positive SedSign group (51.7 ± 8.7 vs 68.4 ± 10.5, *P* < 0.05). The constitutions of clinical diagnosis were significantly different between patients with a positive SedSign and those with a negative SedSign (*P* < 0.001). Overall, nerve roots of the vast majority of patients (48/56, 85.7%) subsided to the ventral side of the dural sac on the prone MR images, although that of 8 (14.3%) patients remain stay in the dorsal side of dural sac. Nerve roots of only one patient with negative SedSign did not settle to the ventral dural sac, while this phenomenon occurred in 7 patients in positive SedSign group (4% vs 22.6%, *P* < 0.001). In addition, the nerve roots of all the five patients subsided to the left side of dural sac on lateral position MR images.

**Conclusions:**

The nerve roots sedimentation followed the direction of gravity. Positive SedSign may be a MR sign of lumbar pathology involved the spinal canal.

## Background

Lumbar spinal stenosis (LSS) is a common cause of back pain and often leads to neurogenic claudication which requires decompression surgery [1–3]. Although medical history, physical examination, and radiologic study are widely used for the diagnosis of LSS, the establishment of a LSS diagnosis lacks a well-accepted standard [4, 5]. An intuitive and objective tool that can facilitate the diagnosis and prognosis of LSS, therefore, is with important clinical significance in clinical practice.

A study by Barz and colleagues first reported that sedimentation sign (SedSign) was helpful in the clinical diagnosis of LSS, as a positive SedSign was common in LSS patients [6]. After this original work, a number of studies consistently suggested that SedSign was a powerful tool to benefit the diagnosis of LSS [7–9]. Such a view, however, was questioned by some researchers that a positive SedSign was merely suggestive of severe LSS [10, 11] and the capability of SedSign in differentiating LSS from non-specific back pain was limited [11–13]. Although the inconsistent findings may be due to patient sampling [11] and varied definitions of stenosis [13, 14], it is important to note that such controversies reflected, more or less, the poor understanding of the mechanism leading to positive SedSign. The spatial status of the nerve roots in the lumbar spinal canal deserves further investigations using subjects in health and disease.

As the SedSign was evaluated on lumbar spine magnetic resonance (MR) images which is conducted in a supine position, Barz postulated that the sedimentation of nerve roots to the dorsal dural sac may be attributable to gravity [6]. Although such a straightforward theory is reasonable, clinical evidence is absent. In other words, it remains unclear whether or not the nerve roots of a health spine would sediment toward the direction of gravity. Given the degree of lumbar lordosis varies in different body positions, mechanical tension the nerve roots undertake may also change in strength and direction. To date, the overall impact of gravity in nerve roots sedimentation in the lumbar canal remains unstudied. The purpose of this study was to determine the role of gravity in the sedimentation of nerve roots in the lumbar canal by MR imaging the lumbar spine in various body positions.

## Methods

### Subjects

We first screened the MR images archive system and identified 734 patients who conducted lumbar spine MR imaging at our hospital between Jan 2018 to Dec 2018. Based on medical record, patients with LSS, disc herniation, non-specific back pain, and spondylolisthesis were selected. Patients with spinal tumors, spinal infections, and spinal deformity, and those who had a history of spine surgery were excluded. After the preliminary screening, 135 patients who met the criteria were included to evaluate SedSign on axial lumbar MR images. All the 135 patients were called back to performing MR imaging in a defined time, and 56 patients have time to finish a follow-up and then included in our study. Based on the presence or absence of SedSign, 31 patients with positive SedSign and another 25 with negative SedSign were selected. The study protocol was introduced to the patients and a written informed consent was obtained for each participant.

### MR imaging

The patients were first subjected to conventional supine MR imaging (SuperScan-1.5 T, Xingaoyi Co., Ltd., Ningbo, China). Each patient was instructed to lay in a standard supine position, with hip and knee joints bent over a wedge cushion. After supine MR imaging, the patient was asked to have 3-min relaxation in standing position and was trained to breathe evenly and slowly for 3 min. Then, the patient was instructed to lay in a prone position upon a surgical foam pad, with the face on a contoured U-shaped pillow. The patient’s lumbar spine was then MR imaged in the prone position. Respiratory gating was used to reduce the interference from breathing. Five patients were selected randomly from the 56 patients and then further MR imaged in left lateral position. Two patients were selected from the negative group and they were diagnosed as non-specific back pain. Another three were from the positive group and they were diagnosed as LSS.

The same protocol was used for supine, prone and lateral MR imaging. T2-weighted (T2W) sagittal and axial images were acquired for the lumbar spine. Axial T2W images were acquired using a turbo spin-echo sequence with a repetition time of 3810 ms, echo time of 120 ms, field of view of 18 cm × 18 cm, slice thickness of 4 mm and slice gap of 0.3 mm. The axial MR imaging was performed parallel to the vertebral endplates and 3 axial images were acquired for each lumbar intervertebral disc, with 2 through the lower and upper vertebral endplates and 1 through the middle of the disc.

### SedSign evaluation

On the T2W axial MR images which were acquired on the supine position, SedSign and morphology grading of lumbar spinal canal were evaluated by two orthopedic surgeons using Barz’s criterion [6]. Briefly, a negative SedSign was defined as nerve roots settled to the dorsal dural sac except for the two exiting nerve roots, and a positive SedSign was considered when the nerve roots suspended or dispersed in the middle of dural sac, or even reached the ventral side of the dural sac [6]. The sedimentation of the nerve roots was evaluated on the axial MR images at the disc level. The SedSign evaluation was first assessed by two authors. We have found high inter-rater agreement (k = 0.85) for the classification of SedSign. When there was any inconsistence in the SedSign evaluation, it was discussed with a senior radiologist together to reach a final rating.

### Classification of nerve roots position in the prone position MR images

The position of nerve roots in lumbar spinal canal was reviewed in all the sagittal and axial MR images in prone position. The patient was classified as “nerve roots settle to the ventral dural sac” if the nerve roots settled to ventral dural sac in all the axial MR images when changed to prone position, while the patient was rated as “nerve roots sequester/stay in the dorsal dural sac” if nerve roots in one section did not sediment to the ventral dural sac on prone position MR images. Two authors, who were blinded to the patients’ information, performed the evaluation independently. The SedSign evaluated in prone position was also performed by the two raters using Barz’s criterion. We also have obtained high inter-rater agreement (k = 0.81) for the classification of SedSign prone position MR images. The final rating was also discussed with a senior radiologist if inconsistent results occurred.

### Statistical analysis

Descriptive statistics were used to depict the characteristics of the patients. T-tests were used to examine the age difference among various groups. Chi-squared tests were used to compare the incidence rates of SedSign among various groups. Statistical analyses were performed using STATA (version 12.0, StataCorp LP, TX, USA).

## Results

### Demographics

There were 56 patients studied, including 31men and 25 women. The mean age was 60.4 ± 12.8 (range 25 to 80 years). Patients with a negative SedSign was younger than those with a positive SedSign (51.6 ± 13 vs 67.5 ± 7, *P* < 0.05, Table 1). Male/female ratios were not different between positive and negative SedSign groups.

**Table 1:** Demographics and clinical diagnosis of patients studied

	Negative SedSign (*N* = 25)	Positive SedSign (*N* = 31)
Age	51.6 ± 13	67.5 ± 7^a^
Male/female	12/13	19/12
Clinical Diagnosis^b^		
Lumbar spinal stenosis	0	17
Lumbar disc herniation	6	12
Spondylolisthesis	1	2
Nonspecific back pain	18	0

^a^ Data were mean ± SD, and t-test was used, *P* < 0.001; ^b^: Chi squared test was used, *P* < 0.001. clinical diagnoses were significantly different between SedSign positive and negative groups

### SedSign and clinical diagnosis

All patients with a positive SedSign had significant MR findings which involved the spinal canal. A positive SedSign occurred in patients with a clinical diagnosis of lumbar disc herniation (12 cases), LSS (17 cases), and spondylolisthesis (2 cases), but not non-specific back pain. For those with a negative SedSign, 18 were clinically diagnosed as having non-specific back pain, 6 with lumbar disc herniation, and 1 with spondylolisthesis (Table 1). The constitutions of clinical diagnosis were significantly different between patients with a positive SedSign and those with a negative SedSign (*P* < 0.001).

### The sedimentation of nerve roots on MR images of various body position

**Fig. 1:**
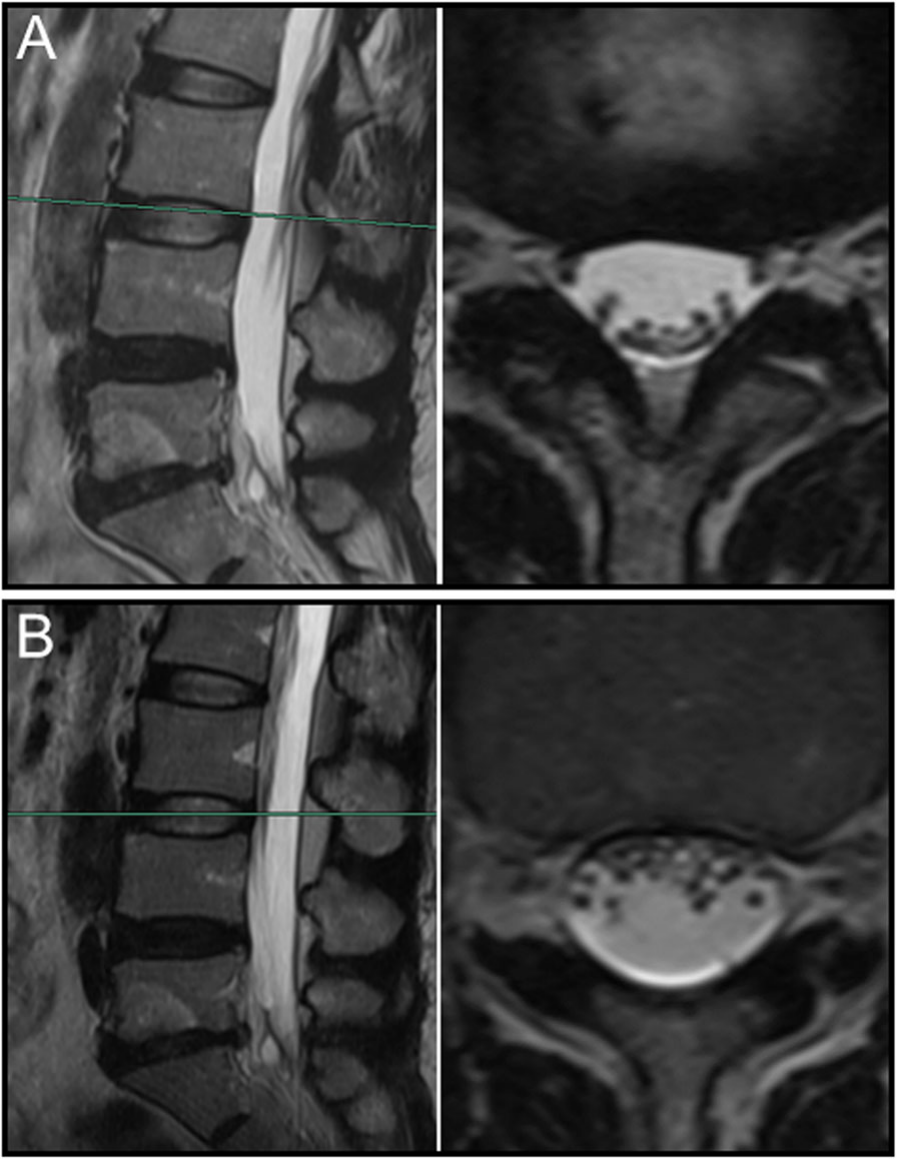
In a woman with negative SedSign (**a**), the nerve roots sedimented to the ventral side of dural sac on prone MR images (**b**)

**Fig. 2:**
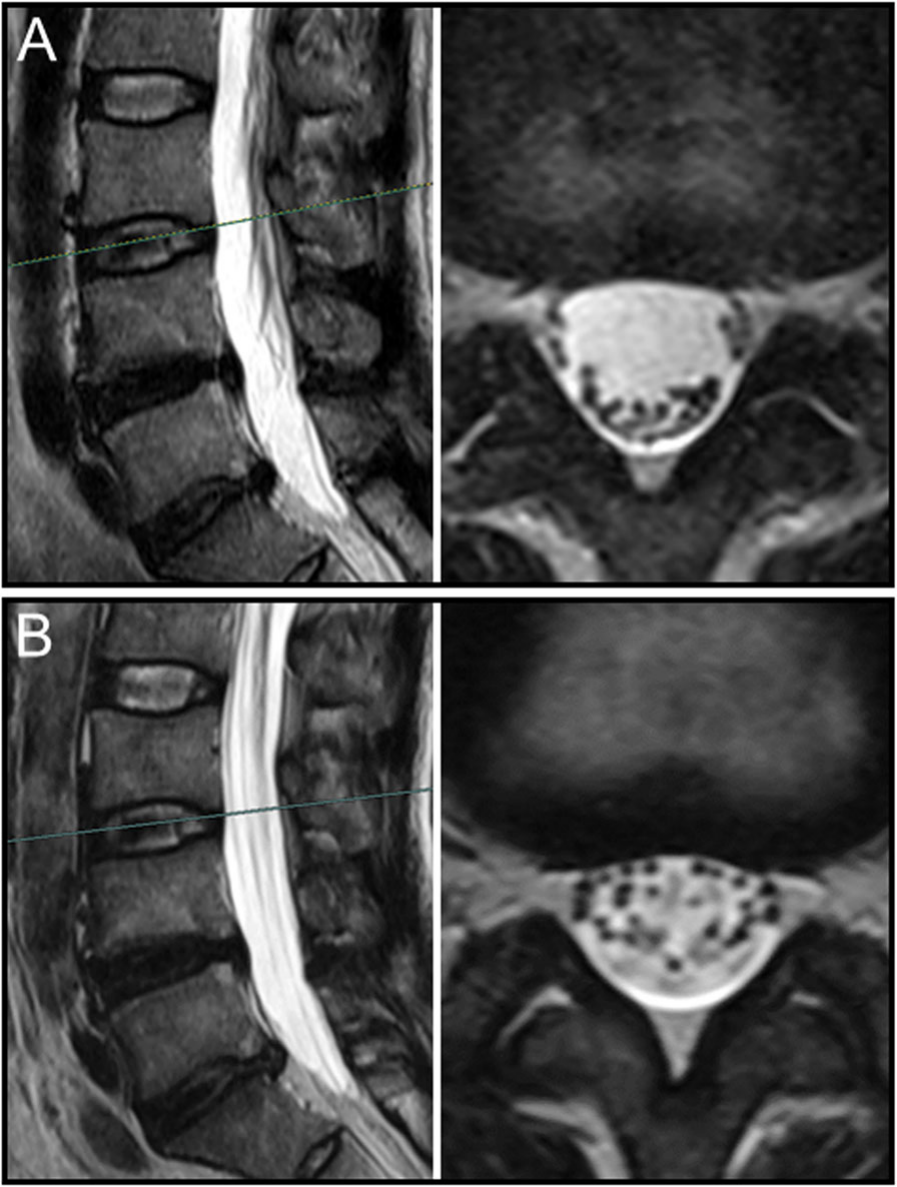
In a man with negative SedSign (**a**), the nerve roots did not subside to the ventral side of dural sac but floating in the middle of dural sac on prone MR images (**b**)

**Fig. 3:**
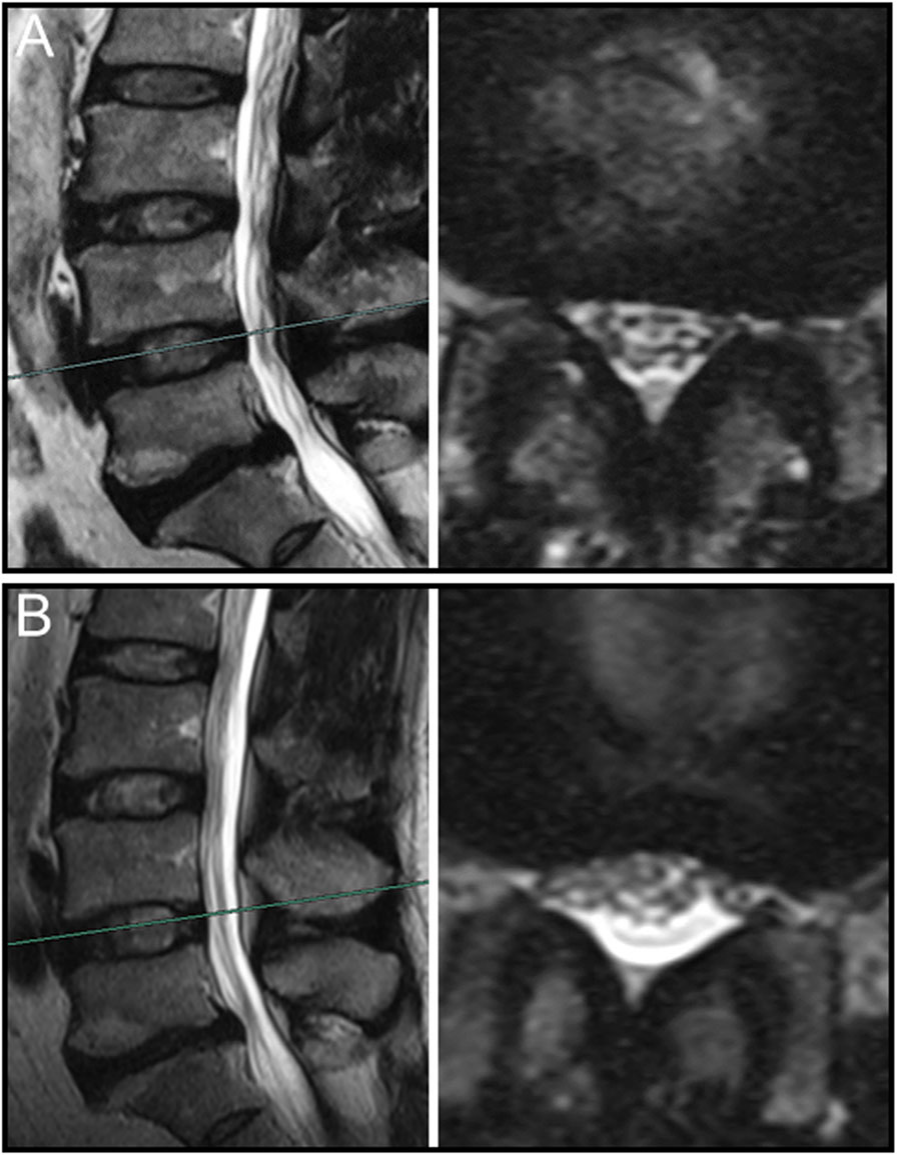
In a woman with positive SedSign (**a**), the nerve roots subsided to the ventral side of dural sac on prone MR images (**b**)

**Fig. 4:**
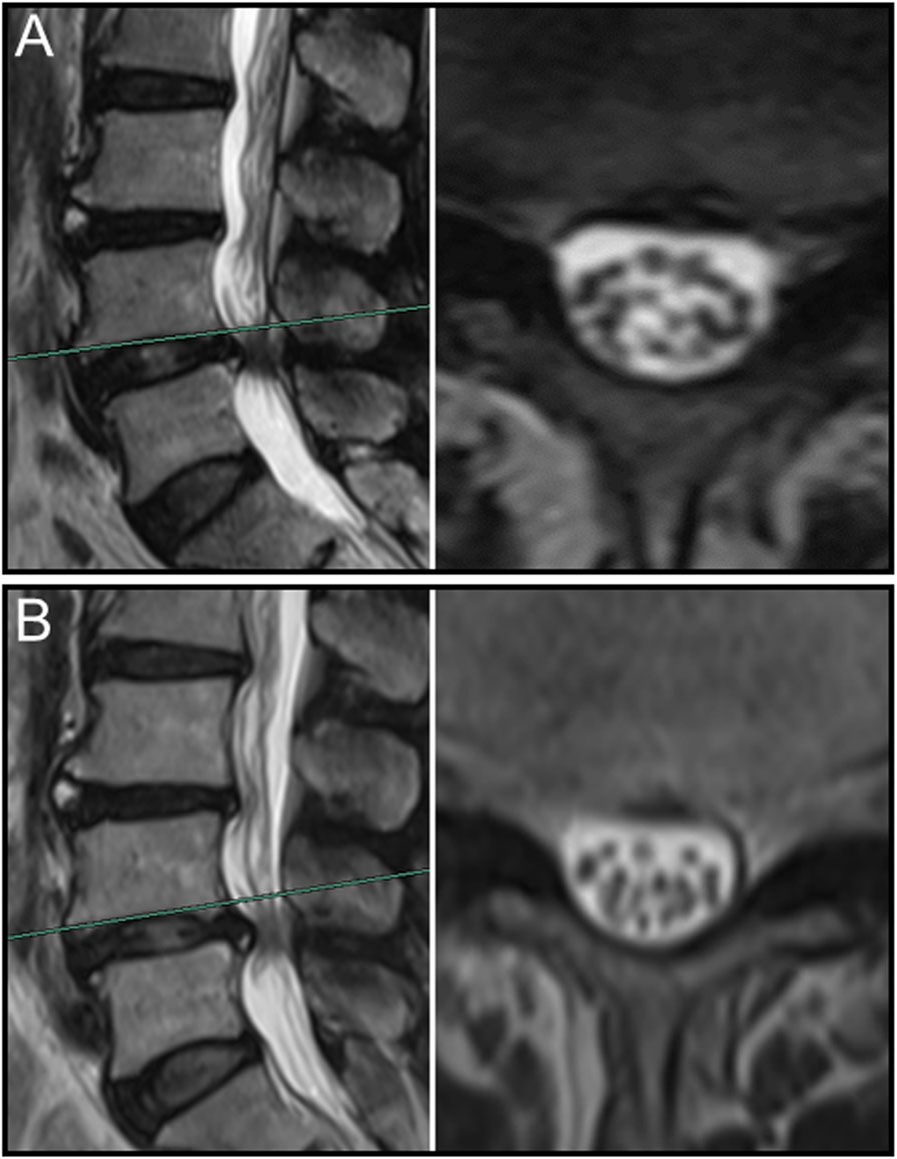
In a woman with positive SedSign and single level spinal stenosis (**a**), the nerve roots remained stay in the dorsal side of dural sac on prone MR images (**b**)

**Fig. 5:**
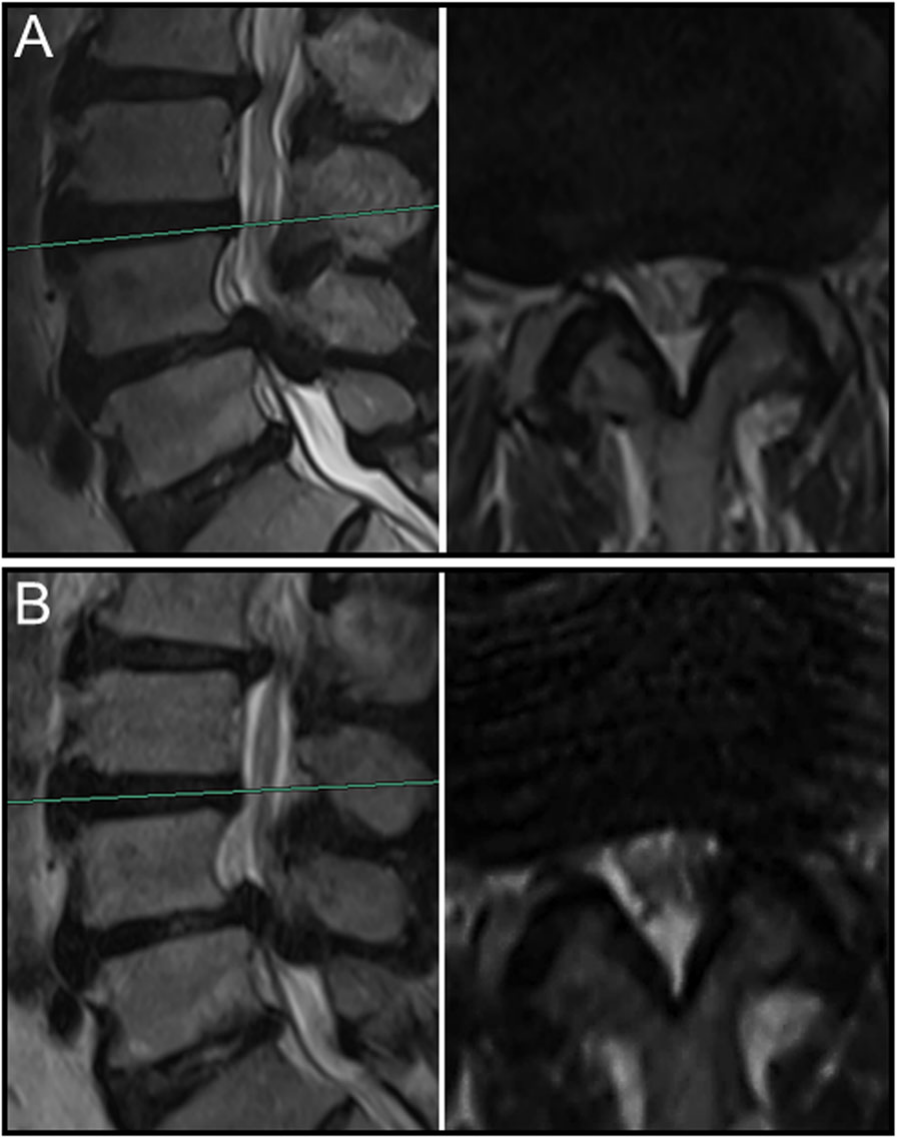
In a man with positive SedSign and multiple level spinal stenosis (**a**), the nerve roots remained stay the ventral side of dural sac on prone MR images (**b**)

**Fig. 6:**
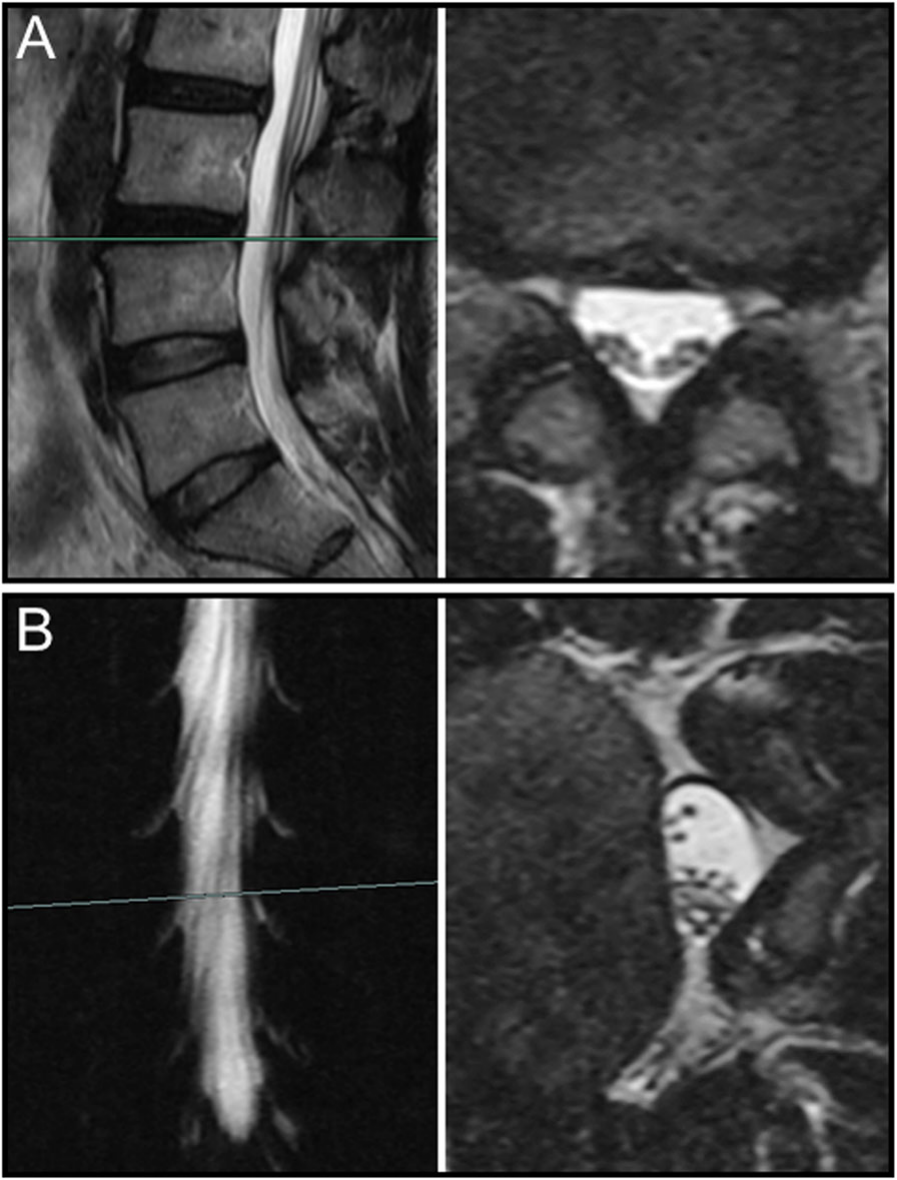
In a man with negative SedSign (**a**), the nerve roots subsided to the left side of dural sac on left lateral position MR images (**b**)

Overall, nerve roots of 48 (85.7%) patients subsided to the ventral side of the dural sac on the prone MR images, although that of 8 (14.3%) patients remain stay in the dorsal side of dural sac. Of the 25 patients with a negative SedSign on supine MR images, the nerve roots settled to the ventral side of the dural sac on prone MR images in 24 patients **(**Fig. 1). Nerve roots in the remaining 1 patient, who was diagnosed as disc herniation, did not settle to the ventral dural sac (Fig. 2). For all the 31 patients with a positive SedSign, nerve roots subsided to the ventral side of the dural sac on the prone MR images in 24 patients (Table 2, Fig. 3). The nerve roots of the other 7 patients still stay (sequester) in the dorsal dural sac, which were due to single level (Fig. 4) or multiple level stenosis (Fig. 5). The nerve roots in patients with positive SedSign were more likely to stay in the dorsal dural sac on prone position MR images (22.6% vs 4%, *P* < 0.05, Table 2). In addition, the nerve roots subsided to the left dural sac in all the 5 patients when they were MR imaged on a left lateral position (Fig. 6).

**Table 2:** Changes of nerve roots sedimentation in supine and prone MR images in patients with both negative and positive SedSign

Supine MR images	Negative SedSign (*N* = 25)	Positive SedSign (*N* = 31)
Prone MR images		
Nerve roots settle to the ventral dural sac	24	24
Nerve roots stay in the dorsal dural sac	1	7

Chi squared test, *P* < 0.001

## Discussion

Our study demonstrated for the first time that gravity determined the direction of nerve roots sedimentation, since nerve roots in the lumbar canal subside along with the direction of gravity on MR images of various positions. In addition, the nerve roots in patients with positive SedSign were more likely to stay in the dorsal dural sac on prone position MR images.

MR imaging, with special sequence, is more and more commonly used in the diagnose of neurological diseases [15]. Recently, diffusion tensor imaging allows for three-dimensional rendering of the peripheral nerves, it was reported that DTI was helpful to determine the extent of neural dysfunction of the carpal tunnel syndrome [16]. Also, MR imaging is useful in evaluation of the spine and helpful in differentiating malignant from benign compressed vertebrae [17]. Through its ability of noninvasive and repeatable measurements, MR imaging offer great help, not only to clinical diagnosis and measure severity of spinal stenosis [12, 18], but also as a tool for basic and clinical research [12, 18].

It is of interest to explore spatial status of the nerve roots in the lumbar spinal canal in different body position and may provide implications for researches of spinal stenosis, since MR imaging is well able to observe the nerve roots due to its high resolution. As clearly demonstrated in the present study, gravity was the major factor determining the direction of nerve roots sedimentation. If the nerve roots do not subside along with the direction of gravity, a positive SedSign on supine MR images for example, there must be an integrated mechanical force (towards the ventral side) against the gravity so that the natural sedimentation of the nerve roots was restricted. Similarly, when changed to prone position, nerve roots of some patients did not settle down the ventral dural sac, and this phenomenon was more likely to occur in patients with disc herniation or spinal stenosis. Since positive nerve root SedSign in prone position was more likely presented in multi-level or severe LSS, it may be an indication of severity of LSS and suggestive of decompressions. Nevertheless, it requires large sample size research combined with the clinical data, such as walking distance and symptom duration.

The mechanism underlying nerve roots floating in the spinal canal against gravity remains unclear. We postulated that such an integrated force against gravity may result from multiple factors, including the length and tension of the nerve roots, the level of spinal stenosis, and the degree of lumbar lordosis. Alterations of these factors, as we clearly see, typically occurred in presence of degenerative changes in lumbar spine. In this study, positive SedSign on prone position MR images occurred mostly in multiple level spinal stenosis. In addition, the nerve roots in those case were tensioned (Fig. 5), which was also observed in the case with negative SedSign (Fig. 2). However, nerve roots did not subside in the direction of gravity despite loosen nerve roots in redundancy (Fig. 4). The role of tension in nerve roots sedimentation can be further studied using patients whose nerve roots tension is high, such as tethered spinal cord syndrome.

Consistent with previous reports [11, 19, 20], our study suggested that a positive SedSign was tended to occur in patients with degenerative lumbar spine disorders which involved the spinal canal, particularly severe LSS. On the other hand, none of the 18 patients with nonspecific back pain presented a positive SedSign. Findings support the view that a positive SedSign has the potential to facilitate differentiating LSS from those without substantial canal involvement. More elegant diagnostic studies with large sample size are needed to further clarify the clinical value of SedSign.

Sample size of the current study was small and the inclusion of selected patients has inherent limitations. As a radiographic study, we did not have detailed clinical data and thus, the role of SedSign in the clinical diagnosis of LSS was not studied. Also, clinical patients with non-specific back pain were used as a convenient control. It is possible that nerve root SedSign may be different in asymptomatic volunteers or other lumbar spinal conditions. Although the SedSign was not evaluated at the vertebral pedicle level next to stenosis, we can observe that nerve roots subsided to ventral portion of dural sac on sagittal images. Axial MR image through the vertebral pedicle should be obtained and used in SedSign evaluation in future study.

In summary, this study revealed that the direction of nerve root sedimentation was mainly determined by gravity. A positive SedSign on supine lumbar spine MR images occurs only when there was an opposite force on the nerve roots against gravity. Such a mechanical force typically resulted from lumbar degenerative changes involving the spinal canal. The clinical value of SedSign deserves further investigations.

## Data Availability

The datasets used in this study are available from the corresponding author on reasonable request.

## Abbreviations

SedSign: sedimentation sign
MR: magnetic resonance
LSS: lumbar spinal stenosis
T2W: T2-weighted

## References

[1] Amundsen T, Weber H, Nordal HJ, Magnaes B, Abdelnoor M, Lilleas F (2000). Lumbar spinal stenosis: conservative or surgical management?: a prospective 10-year study. Spine (Phila Pa 1976).

[2] Mulholland RC (2008). A survey of the "surgical and research" articles in the European spine journal, 2007. Eur Spine J.

[3] Boswell MV, Trescot AM, Datta S, Schultz DM, Hansen HC, Abdi S, Sehgal N, Shah RV, Singh V, Benyamin RM, Patel VB, Buenaventura RM, Colson JD, Cordner HJ, Epter RS, Jasper JF, Dunbar EE, Atluri SL, Bowman RC, Deer TR, Swicegood JR, Staats PS, Smith HS, Burton AW, Kloth DS, Giordano J, Manchikanti L (2007). Interventional techniques: evidence-based practice guidelines in the management of chronic spinal pain. Pain Physician.

[4] Deer T, Sayed D, Michels J, Josephson Y, Li S, Calodney AK (2019). A review of lumbar spinal stenosis with intermittent neurogenic claudication: disease and diagnosis. Pain Med.

[5] Cook CJ, Cook CE, Reiman MP, Joshi AB, Richardson W, Garcia AN (2020). Systematic review of diagnostic accuracy of patient history, clinical findings, and physical tests in the diagnosis of lumbar spinal stenosis. Eur Spine J.

[6] Barz T, Melloh M, Staub LP, Lord SJ, Lange J, Roder CP, Theis JC, Merk HR (2010). Nerve root sedimentation sign: evaluation of a new radiological sign in lumbar spinal stenosis. Spine (Phila Pa 1976).

[7] Barz T, Staub LP, Melloh M, Hamann G, Lord SJ, Chatfield MD, Bossuyt PM, Lange J, Merk HR (2014). Clinical validity of the nerve root sedimentation sign in patients with suspected lumbar spinal stenosis. Spine J Society.

[8] Kim HJ, Lee KY, Kim WC, Oh YS (2011). Clinical value of nerve root sedimentation sign in lumbar spinal stenosis. J Korean Soc Spine Surg.

[9] Wang G, Peng Z, Li J, Song Z, Wang P (2019). Diagnostic performance of the nerve root sedimentation sign in lumbar spinal stenosis: a systematic review and meta-analysis. Neuroradiology.

[10] Piechota M, Krol R, Elias DA, Wawrzynek W, Lekstan A (2019). The nerve root sedimentation sign in diagnosis of lumbar spinal stenosis. Acta Radiol.

[11] Macedo LG, Wang Y, Battie MC (2013). The sedimentation sign for differential diagnosis of lumbar spinal stenosis. Spine (Phila Pa 1976).

[12] Laudato PA, Kulik G, Schizas C (2015). Relationship between sedimentation sign and morphological grade in symptomatic lumbar spinal stenosis. Eur Spine J.

[13] Tomkins-Lane CC, Quint DJ, Gabriel S, Melloh M, Haig AJ (2013). Nerve root sedimentation sign for the diagnosis of lumbar spinal stenosis: reliability, sensitivity, and specificity. Spine (Phila Pa 1976).

[14] Fazal A, Yoo A, Bendo JA (2013). Does the presence of the nerve root sedimentation sign on MRI correlate with the operative level in patients undergoing posterior lumbar decompression for lumbar stenosis?. Spine J.

[15] Gamaleldin O, Donia M, Elsebaie N, Abdelkhalek Abdelrazek A, Rayan T, Khalifa M (2020). Role of fused three-dimensional time-of-flight magnetic resonance angiography and 3-dimensional T2-weighted imaging sequences in neurovascular compression. World Neurosurgery.

[16] Razek A, Shabana A, El Saied T, Alrefey N (2017). Diffusion tensor imaging of mild-moderate carpal tunnel syndrome: correlation with nerve conduction study and clinical tests. Clin Rheumatol.

[17] Razek A, Sherif F (2019). Diagnostic accuracy of diffusion tensor imaging in differentiating malignant from benign compressed vertebrae. Neuroradiology.

[18] Schizas C, Theumann N, Burn A, Tansey R, Wardlaw D, Smith F, Kulik G (2010). Qualitative grading of severity of lumbar spinal stenosis based on the morphology of the dural sac on magnetic resonance images. Spine.

[19] Barz T, Melloh M, Staub LP, Lord SJ, Lange J, Merk HR (2014). Increased intraoperative epidural pressure in lumbar spinal stenosis patients with a positive nerve root sedimentation sign. Eur Spine J.

[20] Zhang L, Chen R, Liu B, Zhang W, Zhu Y, Rong L (2017). The nerve root sedimentation sign for differential diagnosis of lumbar spinal stenosis: a retrospective, consecutive cohort study. Eur Spine J.

